# Diagnostic performance of biomarker S100B and guideline adherence in routine care of mild head trauma

**DOI:** 10.1186/s13049-022-01062-w

**Published:** 2023-01-10

**Authors:** Mohammed Faisal, Tomas Vedin, Marcus Edelhamre, Jakob Lundager Forberg

**Affiliations:** Lund University Faculty of Medicine: Lunds Universitet Medicinska Fakulteten, Lund, Sweden

**Keywords:** Traumatic brain injury, Adherence, S100B, Intracranial hemorrhage, SNC

## Abstract

**Background:**

The Scandinavian Neurotrauma Committee (SNC) has recommended the use of serum S100B as a biomarker for mild low-risk Traumatic brain injuries (TBI). This study aimed to assess the adherence to the SNC guidelines in clinical practice and the diagnostic performance of S100B in patients with TBI. The aims of this study were to examine adherence to the SNC guideline and the diagnostic accuracy of serum protein S100B.

**Methods:**

Data of consecutive patients of 18 years and above who presented to the emergency department (ED) at Helsingborg Hospital with isolated head injuries, were retrieved from hospital records. Patients with multitrauma, follow-up visits, and visits managed by a nurse without physician involvement were excluded.

**Results:**

A total of 1671 patients were included of which 93 (5.6%) had intracranial hemorrhage. CT scans were performed in 62% of patients. S100B was measured in 26% of patients and 30% of all measurements targeted the low-risk mild head injuries indicated by the guideline. S100B's recommended cut-off value (≥ 0.10 µg/L) had a 100% sensitivity, 47% specificity, 10.1% positive predictive value, and 100% negative predictive value—if applied to the target SNC category (SNC 4). If applied to all patients tested, the sensitivity was 93% for traumatic intracranial hemorrhage (TICH). Current ED practices were adherent to the SNC guideline in 55% of patients. Non-adherent practices occurred in 64% of patients with low-risk mild head injuries (SNC4) including overtesting or undertesting of S100B and CT scans.

**Conclusion:**

Adherence to guidelines was low and associated with a higher admission rate than non-adherence practice but no significant increase in missed TICH or death associated with non-adherence to guideline was found. In routine care, we found that the sensitivity and NPV of serum protein S100B was excellent and safely ruled out TICH when measured in the patient category recommended by the guideline. However, measuring serum protein S100B in patients not recommended by the guideline rendered unacceptably low sensitivity with possible missed TICHs as a consequence. To further delineate the magnitude and impact of non-adherence, more studies are needed.

## Introduction

**Fig. 1:**
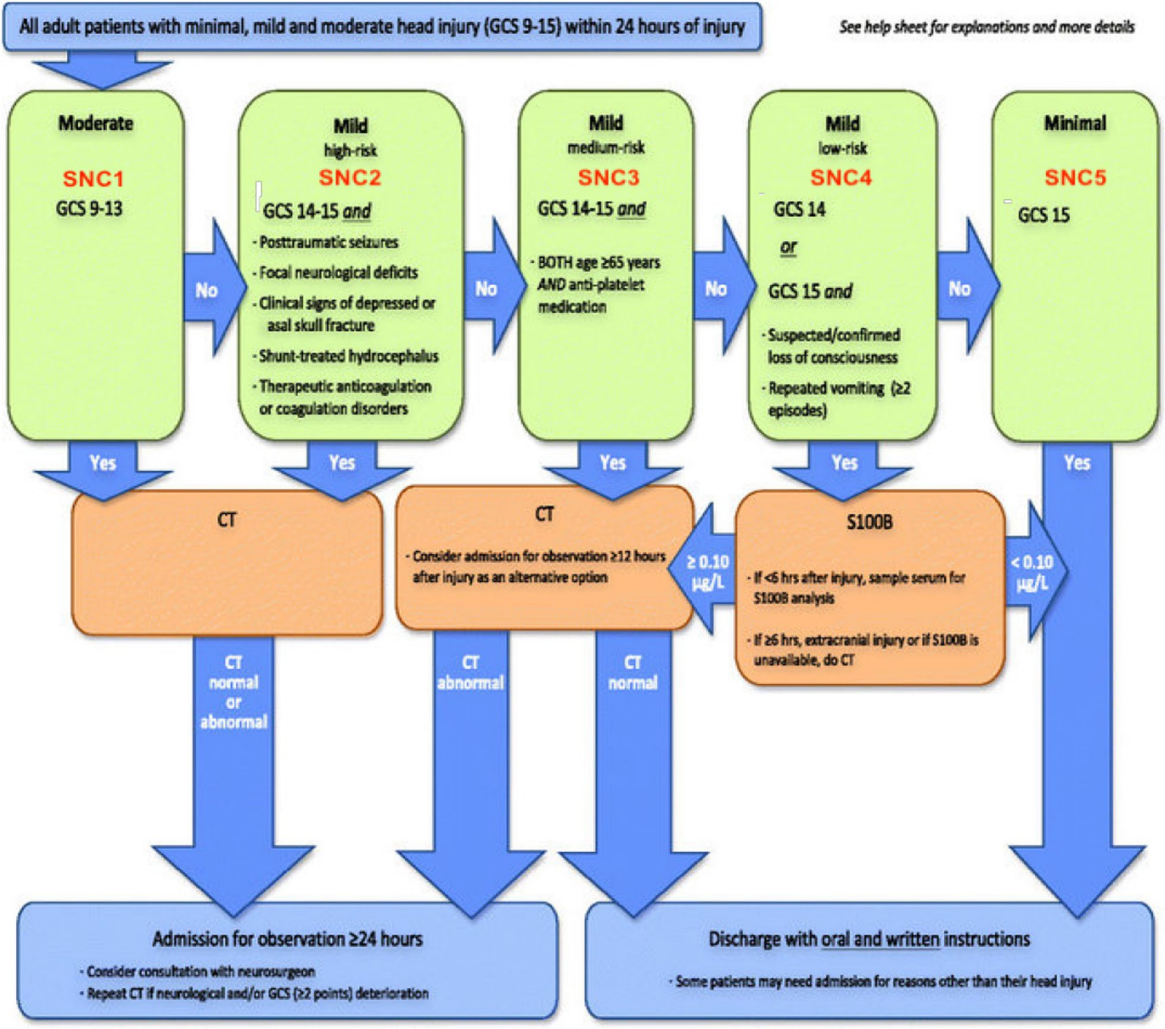
Scandinavian Neurotrauma Committee Guidelines: a name and a number have been added to each category with red color to simplify category referencing throughout the present article (SNC1–5). SNC stands for Scandinavian Neurotrauma Committee. Adapted and published with permission from original author Johan Undén

In Europe, the incidence of traumatic brain injuiry (TBI) is noted at around 300 cases/100 000 people/year [1]. It causes great morbidity, mortality and many emergency department (ED) visits [2]. Moreover, TBI is challenging for ED physicians to manage because signs and symptoms are not always indicative of the extent of brain injury [3, 4]. Because TBI is so common and sometimes difficult to assess medically, adjuncts are needed to aid the clinicians. The gold standard for diagnosing traumatic intracranial hemorrhage (TICH) is computerized tomography (CT) of the head. It is very accurate but disadvantages include exposing the patient to potentially harmful ionizing radiation and a relatively high cost [5–8]. Clinical practice guidelines (CPGs) can help risk stratify and select patients that should undergo a CT-head scan (e.g. Canadian CT Head Rule, National Institute for health and care excellence (NICE) and Scandinavian Neurotrauma Committee (SNC)). Theses algorithms have high negative predictive values (> 99%) but lower specificity (45–60% [9, 10] for detecting TICH requiring neurosurgical intervention. The specificity for TICH might be as low as 34% [11]. However, these numbers are derived from validation studies and theoretical retrospective applications where the guidelines are tested under ideal conditions. Furthermore, the adherence to CPGs in TBI varies widely but adherence to the SNC CPG has been reported at 40–60% [12, 13]. Guideline adherence has been reported to reduce TBI-mortality [12]. The SNC guideline recommends using serum protein S100B level to rule out TICH. Please see Fig. 1 for graphical illustration of the SNC guideline [14]. S100B is the most studied biomarker for ruling out TICH but others exist as well. The clinical cutoff of 0.1 µg/l of serum protein S100B level is set to ensure that no dangerous TICHs are missed and has a negative predictive value of > 99% but a specificity as low as 30–50% [15, 16]. It is only recommended in SNC category 4 and only as a “rule-out” test. The low specificity is of little importance in this category because if no S100B-test was available, all patients would be prescribed a head-CT.

The primary aim of this study was to investigate the adherence to the Scandinavian Neurotrauma Committee head trauma guidelines. The secondary aim was to assess the diagnostic accuracy of brain biomarker serum protein S100B.

## Material and methods

Data on consecutive patients presenting to the ED at Helsingborg General Hospital with isolated head injury was collected retrospectively through medical records. Data collection was performed in patients registered between January 1, 2017, and December 31, 2017.

The hospital provides secondary care for 300,000 people which generates 70,000 ED visits per annum. Tertiary neurosurgical care is provided at Skane’s University Hospital, 40 km away. Multitrauma patients were managed according to ATLS™. The in-hospital guideline for traumatic brain injury during the study period was the SNC guideline [14].

The inclusion criteria were adult patients (≥ 18 years) attending the ED with “head trauma” as the chief complaint. Exclusion criteria were multitrauma, follow-up visits, visits managed by a nurse without physician’s involvement and confidential medical records. Some included patients had additional minor injuries, however, all patients triaged as multitrauma (n = 647) were excluded. The multitrauma definition used was in accordance with the 2014 Berlin definition [17]. This was done to ensure that the cohort was representative of ED patients with minor traumatic brain injury that are managed according to a head trauma CPG.

To make results of the present study clear and easy to understand, a modification of the original SNC flow chart has been made where a name and a number have been added to each risk category (SNC1–5). This is shown in Fig. 1.

The primary aim (adherence) was assessed in all SNC categories (Categories 1–5) and outcome measures were number of CTs, serum protein S100B assays, admissions, neurosurgical interventions and deaths. The secondary aim (diagnostic accuracy of serum protein S100B level) was reported for SNC category 4 separately and for all S100B-measurements together. The purpose of this was to outline how diagnostic accuracy was affected by S100B-measurement not indicated by the guideline. Outcome measures for secondary aim are further described in “statistical analysis” below.

To make assessment of level of consciousness internationally valid, it was converted from Reaction Level Scale (RLS) to Glasgow Coma Scale (GCS) and reported as GCS throughout the study. Earlier articles have reported good correlation between RLS1–2 and GCS 14–15 but differences between RLS3 and GCS13–8. Because of this, level of consciousness was only reported as GCS15-14 and GCS < 14 [18, 19]. Loss of consciousness was defined as any length of loss of partial or complete loss of perception of oneself and/or the surroundings.

Guidelines for retrospective reviews developed by Vassar and Holzmann [20] were followed.

### Statistical analysis

Data was analyzed with SPSS version 25 for Mac. Histograms and Shapiro-Wilks formula were used to test for normal distribution. Statistical significance was set to *p* < 0.05. Central tendencies were presented as medians with interquartile range when non-parametric. Descriptive statistics were used to describe the material. Serum protein S100B level diagnostic accuracy was evaluated with sensitivity, specificity, negative predictive value, positive predictive value and Receiver operator Characteristics (ROC) curve with area under the curve (AUC) assessment. Contingency tables were tested using the χ^2^ test or Fisher’s test when applicable.

## Results

**Fig. 2:**
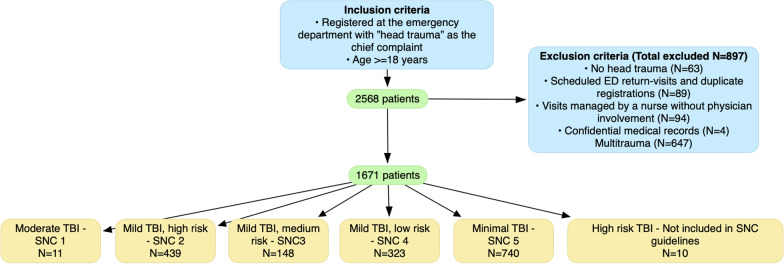
ROC curve of S100B for predicting intracranial hemorrhage following traumatic head injuries. **A** Patients in which SNC guideline recommend S100B testing (SNC4/Mild, Low risk), (n = 131). **B** All patients with S100B measurements (n = 434)

This study included 1671 patients with head injuries with a median age of 64 years (interquartile range 39–80), and 47% were females. See Fig. 2 for inclusion process and distribution of different SNC categories. Other demographic, clinical and laboratory characteristics of the studied patients are summarized in Table 1. Head injury was minimal (SNC5) in 44.3%, mild (SNC2–4) in 54.5%, and moderate (SNC1) in 0.7% of all patients. Ten patients had severe head injuries and were therefore not classified according to SNC. The proportion of patients admitted to the hospital, received neurosurgical interventions, or died increased with the severity of head injuries (see Table 2). CT-scans were performed in 1039 patients (62.2%), serum protein S100B level was assessed in 434 patients (26.0%) of which 131 where in the recommended SNC category 4. CT scan detected intracranial hemorrhage (ICH) in 93 patients (5.6%; 95% confidence interval (CI) 4.5–6.7%). Of these 93 ICHs, 27 were subdural, 2 were epidural, 12 were categorized as subarachnoid, 8 were contusions and 44 were not clearly described in the radiology report.

**Table 1:** Demographic, clinical and laboratory characteristics of the studied population

Variables	No. (% of N = 1671)
Age (years), median (interquartile range)	64.0 (39–80)
< 65	846 (50.6%)
≥ 65	825 (49.4%)
Gender	
Female	784 (46.9%)
Male	887 (53.1%)
Medical history	
Bleeding disorders	10 (0.6%)
Thrombocyte-inhibitors medications	194 (11.6%)
Oral anticoagulant medications	215 (12.9%)
LMW Heparin treatment	6 (0.4%)
High-energy trauma (according to ATLS)	8 (0.5%)
Clinical presentations	
Loss of consciousness	446 (26.7%)
Amnesia	378 (22.6%)
Headache	388 (23.2%)
Worsening headache	67 (4.0%)
Vomiting	109 (6.5%)
Posttraumatic seizures	20 (1.2%)
Alcohol/drug intoxication	385 (23.0%)
Abnormal behavior in emergency department	59 (3.5%)
Signs of depressed/open skull fracture	115 (6.9%)
Signs of basal skull fracture	213 (12.7%)
Signs of facial fracture	253 (15.1%)
Scalp hematoma	80 (4.8%)
Other signs of trauma above the clavicles	1198 (71.7%)
New neurological deficits	86 (5.1%)
GCS score	
> 13	1650 (98.7%)
< 14	21 (1.3%)
CT head and spine findings	
Intracranial hemorrhage (ICH)	93 (5.6%)
Skull fracture	54 (3.2%)
Facial fracture	100 (6.0%)
Cervical spine fracture	16 (1.0%)
Outcomes	
Hospital admission	388 (23.2%)
Neurosurgical intervention	10 (0.6%)
Death	8 (0.5%)

*LMW* low molecular weight, *ATLS* advanced trauma life support

**Table 2:** Distribution of studied patients by S100B measurement, CT scan performed and TICH diagnosis across the SNC guideline categories

Severity of head injury (SNC category)	N	No. (% of N)					
		S100B	CT	TICH	Hospital admission	Neurosurgical intervention	Deaths
Severe (no category)	10	1 (10.0%)	10 (100.0%)	7 (70.0%)	9 (90.0%)	4 (40.0%)	4 (40.0%)
Moderate (SNC 1)	11	2 (18.2%)	11 (100.0%)	5 (45.5%)	8 (72.7%)	2 (18.2%)	1 (9.1%)
Mild, high-risk (SNC 2)	439	87 (19.8%)	361 (82.2%)	30 (6.8%)	151 (34.4%)	2 (0.5%)	2 (0.5%)
Mild, medium-risk (SNC 3)	148	18 (12.2%)	138 (93.2%)	16 (10.8%)	53 (35.8%)	1 (0.7%)	1 (0.7%)
Mild, low-risk (SNC 4)	323	131 (40.6%)	229 (70.9%)	24 (7.4%)	70 (21.7%)	0	0
Minimal (SNC 5)	740	195 (26.4%)	290 (39.2%)	11 (1.5%)	97 (13.1%)	0	0
Total	1671	434 (26.0%)	1039 (62.2%)	93 (5.6%)	388 (23.2%)	10 (0.6%)	8 (0.5%)

*SNC* Scandinavian Neurotrauma Committee, *CT* Computed Tomography, *TICH* Traumatic Intracranial Hemorrhage

In the SNC Category 4 of 323 patients, 229 (70.9%) underwent a head-CT and 24 (7.4%) had ICHs. See Table 2 for information on number of investigations and outcomes.

### Adherence

Current ED practices were adherent to the SNC guideline in 912 (54.6%) patients with regards to head-CTs, serum protein S100B level assays and admissions/discharges. CTs and S100B-assays were prescribed in accordance with the guideline in 77.8% and 77.8% of cases, respectively. Non-adherence existed in 51.1% the patients with minimal head injuries (SNC5), 63.8% of patients with low-risk mild head injuries (SNC4), and 28.8% of patients with higher risk for ICH (SNC0–3). Non-adherent practices included overtesting and undertesting of S100B and CT scan as described in Table 3.

**Table 3:** Distribution of head injury cases by types of non-adherence practices to the SNC guidelines

	N	Overtesting* no			Undertesting** no		Overall no. (% of n)
		S100B	CT	S100B & CT	S100B	CT	
SNC1–3	608	108	-	-	-	88	175 (28.8%)
SNC4	323	-	10	-	192	4	206 (63.8%)
SNC5	740	88	183	107	-	-	378 (51.1%)
Total	1671	196	193	107	192	71	759 (45.4%)

*SNC* Scandinavian Neurotrauma Committee, *CT* computerized tomography

*Investigation not recommended by the SNC guideline but still performed

**Investigation recommended by the SNC guideline but not performed

Adherent and non-adherent practices resulted in 214 (55.2%) and 174 admissions (44.8%), respectively. A total of 147 admissions (37.9% of all hospital admissions, most of them in SNC4 and SNC5 categories) were not indicated by the guideline. Adherence to SNC guideline resulted in more admissions compared to non-adherence (p = 0.011). No statistically significant differences were seen in missed admissions, neurosurgical interventions and deaths (Table 4).

**Table 4:** Distribution of outcomes of head injuries by the adherence to Scandinavian Neurotrauma Committee guideline

Outcomes	Adherence to SNC guideline no. (% of total)			*p*-value
	Total	Non-adherent	Adherent	
Total sample	1671	759 (45.4%)	912 (54.6%)	–
Hospital admissions				
Negative for TICH	305	147 (48.2%)	158 (51.8%)	
Positive for TICH	83	27 (32.5%)	56 (67.5%)	
Total	388	174 (44.8%)	214 (55.2%)	0.011*
Missed admissions	10	3 (30.0%)	7 (70.0%)	0.363^f^
Neurosurgical interventions	10	3 (30.0%)	7 (70.0%)	0.363^f^
Deaths	8	1 (12.5%)	7 (87.5%)	0.079^f^

*TICH* traumatic intracranial hemorrhage

*Statistically significant p-value, ^f^Fisher’s exact test

### Serum protein S100B level assay

In the SNC4 category, 41% of the patients had an assay of serum protein S100B level. The S100B measurements in the SNC4 category represented 30% of all S100 measurements (all categories). S100B measurements were higher or equal to 0.10 µg/L (cut-off value) in 261 patients (60.1%).

**Fig. 3:**
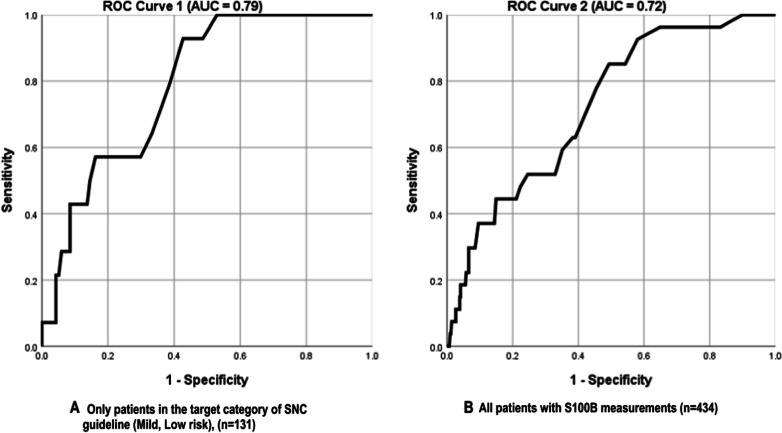
Distributions of S100B measurement, CT scans and overall ED practices to TBI patients according to adherence to SCN guidelines (N = 1671)

Evaluation of the performance of S100B's cut-off value (≥ 0.10 µg/L) in SNC category 4 yielded a sensitivity of 100% (95% CI 76.8—100.0), a specificity of 47% (95% CI 37.7—56.5), a PPV of 10.1% (95% CI 8.6—11.7), and a NPV of 100%. ROC curve analysis of S100B measurements in SNC category 4 had an AUC of 0.79 (95% CI 0.71—0.86; p < 0.001) (Fig. 3A).

However, assay of S100B applied to any patient with TBI regardless of SNC classification, reduced the accuracy; the sensitivity was 93% (95% CI 75.7—99.1); specificity was 42% (95% CI 37.2—47.0); PPV was 8.7% (95% CI 7.6–9.8), and NPV was 99.0% (95% CI 96.2–99.7). If applied to all patients regardless of the SNC classification, the ROC curve showed an AUC of 0.72 (95% CI 0.63 − 0.81; p < 0.001) and similar optimal cut-off value of 0.11 µg/L (Fig. 3B).

## Discussion

This was a retrospective review of the medical records of 1671 TBI patients to investigate the extent of the adherence to the SNC guidelines in real-life settings and the diagnostic accuracy of serum protein S100B level in TBI-patients. The results showed an adherence to SNC guidelines of 54.6%; the non-adherence was concentrated in patients with minimal and low-risk head injuries (SNC 5 and 4) [21].

Such findings run in line with previous reports that noted a substantial discrepancy between guidelines' recommendations and initial management of TBIs in real-life practice. In a recent systematic review, Cnossen et al. [22], noted that the adherence to SNC recommendations in real-life practice ranged between 50 and 60% only. According to their results, non-adherence was strongly associated with prolonged hospitalization. In two reports by Heskestad et al. [23, 24], the rate of non-adherence to SNC recommendations ranged from 50 to 63%; the majority of non-compliance was reported amongst patients with minimal and low-risk head injuries (SNC 5 and 4), and this was mainly in the form of performing unnecessary CT scans. The non-adherence led to over-triage and unnecessary hospital admissions. Another report found that the rate of non-compliance to SNC was 54.5%, 45.1%, and 2.2% in patients with minimal, mild, and moderate TBIs, respectively [25]. Several reasons can contribute to limited compliance to SNC guidelines in a real-life setting. Firstly, insufficient knowledge and misinterpretation of the guidelines may lead physicians to rely on their clinical judgment and experience [14]. Besides, many physicians may consider S100B measurement as a part of routine investigations for patients with suspected TBIs rather than considering it as a valid screening tool for assessing a selected patient group (e.g. SNC4) need for further investigations. The crowded and busy nature of the emergency department may also lead the physicians to seek rapid patient turnover without waiting for laboratory results or ordering biomarkers before risk-stratifying into SNC categories.

In a recent qualitative study on barriers to SNC guideline adherence, interviewees stated that the guideline was useful but that the most important measure to increase adherence would be to increase digital and physical availability of the guideline. Other factors included more concise, easily-read and well-illustrated guidelines as well as a culture that better promoted guideline utilization [26].

Serum S100B can play an important role in predicting patients with TICH who present to the ED with mild TBI and have a low-risk profile; hence, it can reduce the number of unnecessary CT scans [27]. In the present study, we provided real-world evidence that S100B is a useful biomarker for prediction of TICH in mild, low-risk, TBI patients when measured in accordance with present guidelines. At the current cut-off value of > 0.10 μg/l, serum protein S100B had an exccellent sensitivity (100.0%) and negative predictive value (NPV; 100.0%). In line with our findings, Jones et al. [28], demonstrated that the S100B had a NPV of 97.3% for ruling out ICH in patients with mild TBIs. Another recent report showed that the S100B had a 97% sensitivity and 92% NPV in patients with mild TBI [29]. Such findings were consistent with other recent reports [30–32]. In a previous systematic review and meta-analysis on twelve studies, Undén and Romner [27] reported that the serum S100B had a NPV of 99% for detection of TICH in patients with mild TBIs. However, we found that the sensitivity of S100B was lowered to an unacceptable level (93%) in routine clinical practice if not used according to SNC recommendations. Thus, it is very important to risk stratify TBI before using serum protein S100B, otherwise TICHs could potentially be missed.

On the other hand, we found that the serum S100B had a low specificity (47.0%) for detection of TICH in patients with mild TBIs, highlighting that serum S100B has limited utility as a single biomarker for TICH and cannot be used as a rule-in biomarker. In agreement with our findings, Stein et al. [33], reported that the serum S100B had a specificity of 53% in patients with mild TBIs. Thus, to minimize the false-positive results and unnecessary CT scans—particularly in patients with dark skin [34], the decision to perform S100B assay should be combined with clinical evaluation as it is not suited as a TBI-screening tool. Besides, the usefulness of additional investigations, such as electroencephalogram (EEG) and other blood markers [35], could be evaluated in future studies to improve the specificity of serum S100B in initial triage of patients with TBI.

While the present study poses additional insights concerning the adherence to SNC recommendations in real-life setting, we acknowledge the existence of certain limitations.

The choice to exclude multitrauma patients was done because the SNC guideline is not applicable in this subset of patients. The retrospective method has some limitations. Information bias occurs when reviewing medical records and handling missing data. Irrespective of our attempts to prevent this, only careful conclusions can be drawn from this study. The pragmatic way of handling missing data was deliberated in our study group and regarded as the best solution. Nevertheless, it precludes reliability measurements (e.g., confidence intervals) and direction of any bias cannot be quantified. Interpreting “Head-CT not performed” as the “no traumatic intracranial hemorrhage” can entail missed hemorrhages. However, because of the follow-up search for other ED-visits 6 months after the TBI it can be assumed that intracranial hemorrhages with severe consequences would have been found.

Besides, we did not correlate adherence rate with uneventful hospitalized patients or complication rates among discharged patients to reflect the impact of non-adherence to the SNC guideline on the clinical course of TBIs patients.

In conclusion, adherence to guidelines was low and associated with a higher admission rate than non-adherence practice but no significant increase in missed ICH or death associated with non-adherence to guideline was found. In routine care, we found that the sensitivity and NPV of serum protein S100B was excellent and safely ruled out TICH when measured in the patient category recommended by the guideline. However, measuring serum protein S100B in patients not recommended by the guideline rendered unacceptably low sensitivity with possible missed TICHs as a consequence. To further delineate the magnitude and impact of non-adherence, more studies are needed.

## Data Availability

Data will be made available upon reasonable request.

## Abbreviations

AUC: Area under curve
CT: Computed tomography
ED: Emergency department
EEG: Electroencephalogram
ICH: Intracranial hemorrhage
NPV: Negative predictive value
RLS: Reaction Level Scale
ROC: Receiver operator Characteristics
SNC: Scandinavian Neurotrauma Committee
TICH: Traumatic intracranial hemorrhage
TBI: Traumatic brain injuries
